# An experimental investigation on the speed of sand flow through a fixed porous bed

**DOI:** 10.1038/s41598-017-00082-2

**Published:** 2017-03-03

**Authors:** Wanghua Sui, Yankun Liang, Xinjia Zhang, Ravi Jain, Tao Zhu

**Affiliations:** 1 School of Resources and Geosciences, China University of Mining and Technology, Xuzhou, Jiangsu China; 2 School of Engineering and Computer Science, University of the Pacific, California, USA; 3 WanliCoalmine No. 1, Shenhua Shendong Group Co. Ltd., Ordos, Inner Mongolia China

## Abstract

In this study, we document several experiments that investigate the speed of the flow of fine sand through a fixed porous bed of packed glass beads under various conditions, including the height of the sand column (*H*) and porous bed (*h*) and the diameter of the glass beads (*D*) and sand grains (*d*). The experiments are conducted with glass beads packed at a constant density and sand at a different dry bulk density. The results show that the height of the sand does not affect the speed of the sand flow. The speed of the sand flow (*v*) decreases with an increase in *h* until *h* approaches a certain value. An equation ${\boldsymbol{v}}={\boldsymbol{a}}\sqrt{{\bf{g}}{\boldsymbol{D}}}{\bf{l}}{\bf{n}}(\frac{{\boldsymbol{D}}}{{\boldsymbol{kd}}})$ is proposed, where *a* and *k* are the experimentally determined coefficients. Moreover, the flow of sand through a fixed porous bed could be regarded as parallel flow through multiple pipes, and therefore, the relationship between *D* and the number and diameter of pipes, *N* and *D*_p_, are discussed. Further investigations are needed for the result that the flow of sand through a porous bed or multiple parallel pipes cannot be simplified to flow through one orifice with a certain diameter.

## Introduction

Granular materials, such as sand, gravel, rice and sugar, have an important role in physics, chemistry, geology and industrial technology. However, granular matter is very complex; a granular material may behave like a solid or a fluid^1–5^. For instance, a pile of sand can stand tall and fixed like a solid, but sand can also pour through an orifice like liquid. This unique phenomenon has received considerable attention, and much work has been devoted to understanding the fundamental differences between fluid and granular flows. Regardless of the medium through which fluid flows through, the speed of the flow is proportional to the water head (or liquid pressure) difference, for instance, as demonstrated by the well known Darcy’s law for groundwater seepage. However, the force exerted by the head of the granular material minimally affects the speed of flow when the granular matter flows through a silo or an orifice^6–12^. The widely accepted Beverloo’s law shows that the flow rate of grains through orifices depends on the diameter to a 5/2 power law. Research work have shown that even though Beverloo’s correlation fits very well to the flow rate for large orifices, it fails for small orifices where clogging could take place^13–15^. This has been experimentally validated by Mankoc *et al*.^16, 17^.

As a classical and conventional issue in granular dynamics, the flow of sand through an orifice has been the topic of active discussion. However, there is little published work on how small particles move through fixed large particles even only under the condition of gravity. In western China, a new type of geological hazard has taken place due to mining under thin bedrock and aeolian sand. Sand flows through the fracture and caving zones into underground panels and cause safety concerns^18, 19^ (see Supplementary Information for a detailed example of this scenario in a coalmine). There has been a lack of research that examines the characteristics of sand flow through porous granular media; a research gap which this study intends to address. In this study, several experiments are documented to show how fine sand flows through a fixed porous bed. The results are helpful for predicting the speed of the grain flow through porous medium in the industry or determining the possibility of geological hazards.

## Results

**Figure 1:**
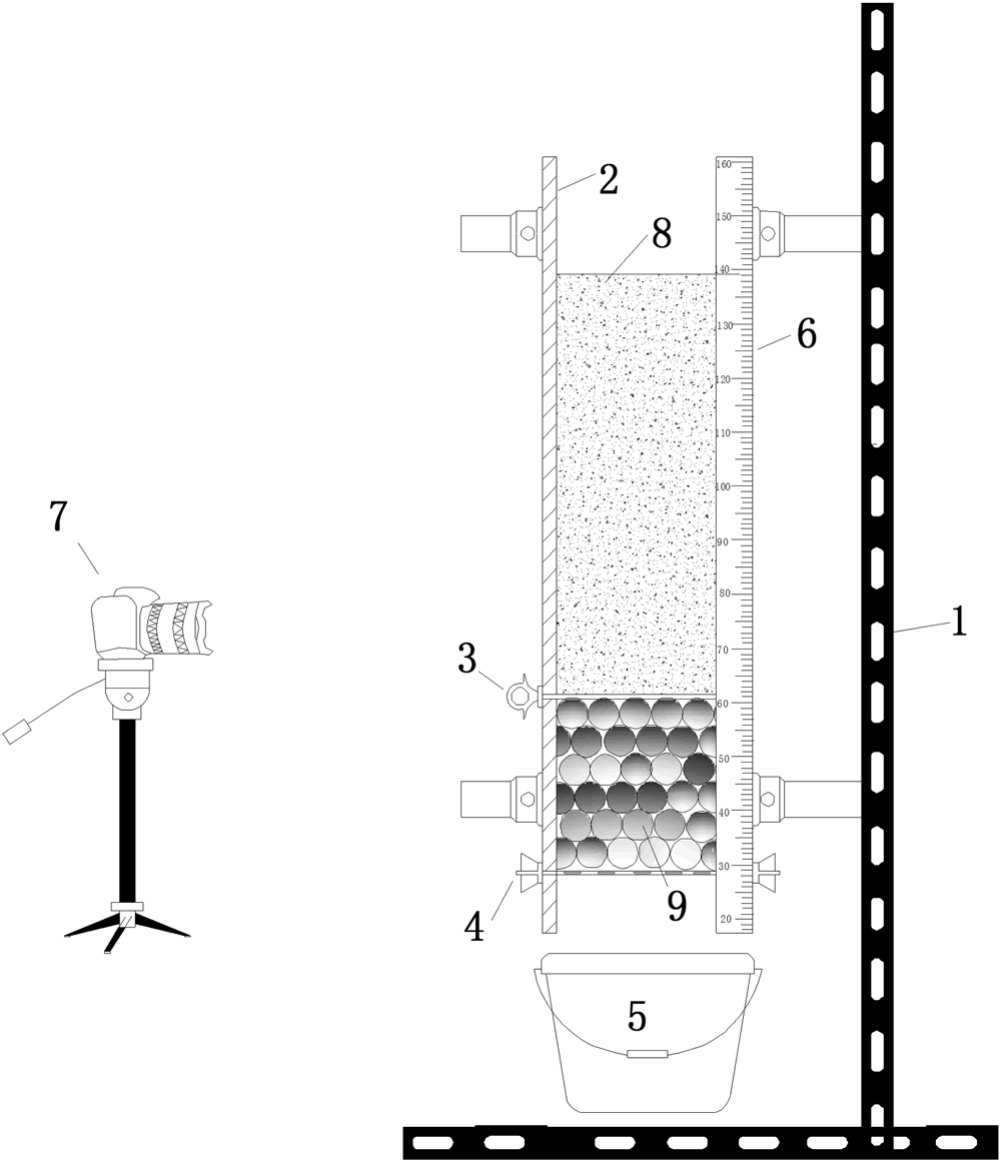
Experimental set up. 1- Bracket; 2- Cylindrical tube; 3- Bulkhead; 4- Chicken wire; 5- Bucket; 6- Scale; 7- High speed camera; 8 - Sand; 9 - Glass beads.

The experiments were conducted in a cylindrical tube with a height of 2000 mm and a diameter of 120 mm. Dry sand and glass beads were used as the two different types of granular matter. Same sized glass beads that were placed at the bottom of the cylindrical tube represented the fixed porous bed. A plastic plate was placed on the top surface of the glass beads, so that the sand would flow instantaneously through them when the plate was removed for the commencement of the experiment, as shown in Fig. 1.

### Characteristics of sand flow through porous bed

**Figure 2:**
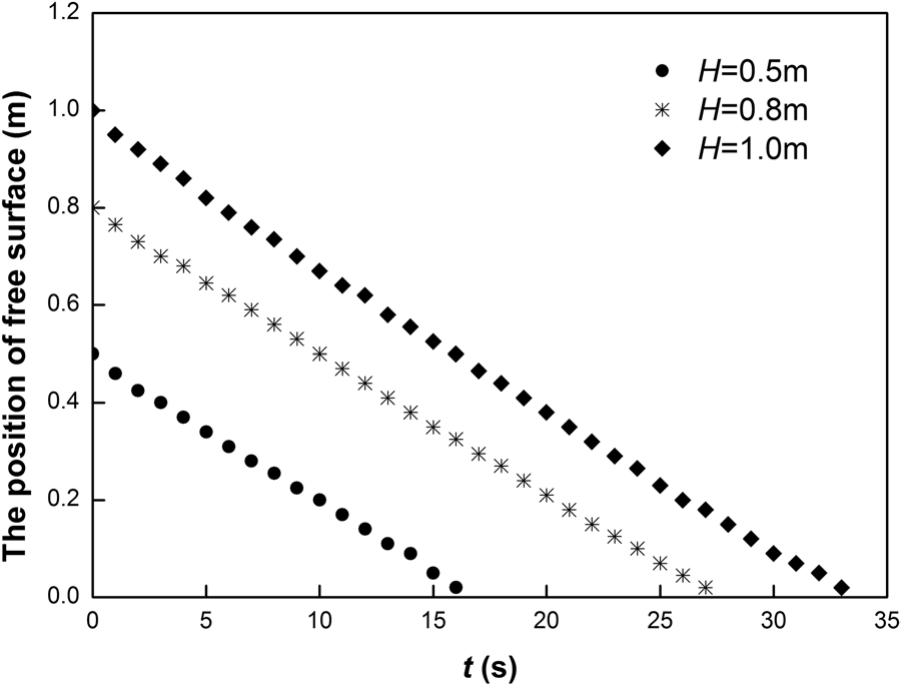
Position of free surface versus time under different heights of sand column (*H*).

In the modeling, the sand was compacted to a certain height. The experiments commenced with the removal of the bulkhead. During the experiment, the dropping top surface of the dry sand was always flat. In this case, the speed of the sand flow is expressed by using the drop distance of the sand per second. It was found that the speed *v* is constant for different heights of the sand column (*H* = 0.5, 0.8, and 1.0 m). Figure 2 shows the position of the free surface versus time, where the height of the porous bed *h*, diameter of the glass beads *D*, and range of the grain size of the sand *d* are 105 mm, 21 mm and 0.1 to 0.5 mm, respectively. The speed *v* is approximately 33.4 mm/s under these conditions. It is noted that the speed of the sand flow is independent of the initial height of the sand above the fixed porous bed. This confirms that the height of the granular matter does not affect the speed of the flow, even with flow through a porous bed.

### Influential factors of sand flow

The results showed that the speed of the sand flow under the existing gravity flow is related to the height of the porous bed (*h*), diameter of the glass beads (*D)* and the diameter of the sand grains (*d*). The influence of *h*, *D* and *d* on the sand flow was experimentally investigated, respectively (see Method section). Corresponding videos are provided in the Supplementary Information as S1 to S3, respectively.

**Figure 3:**
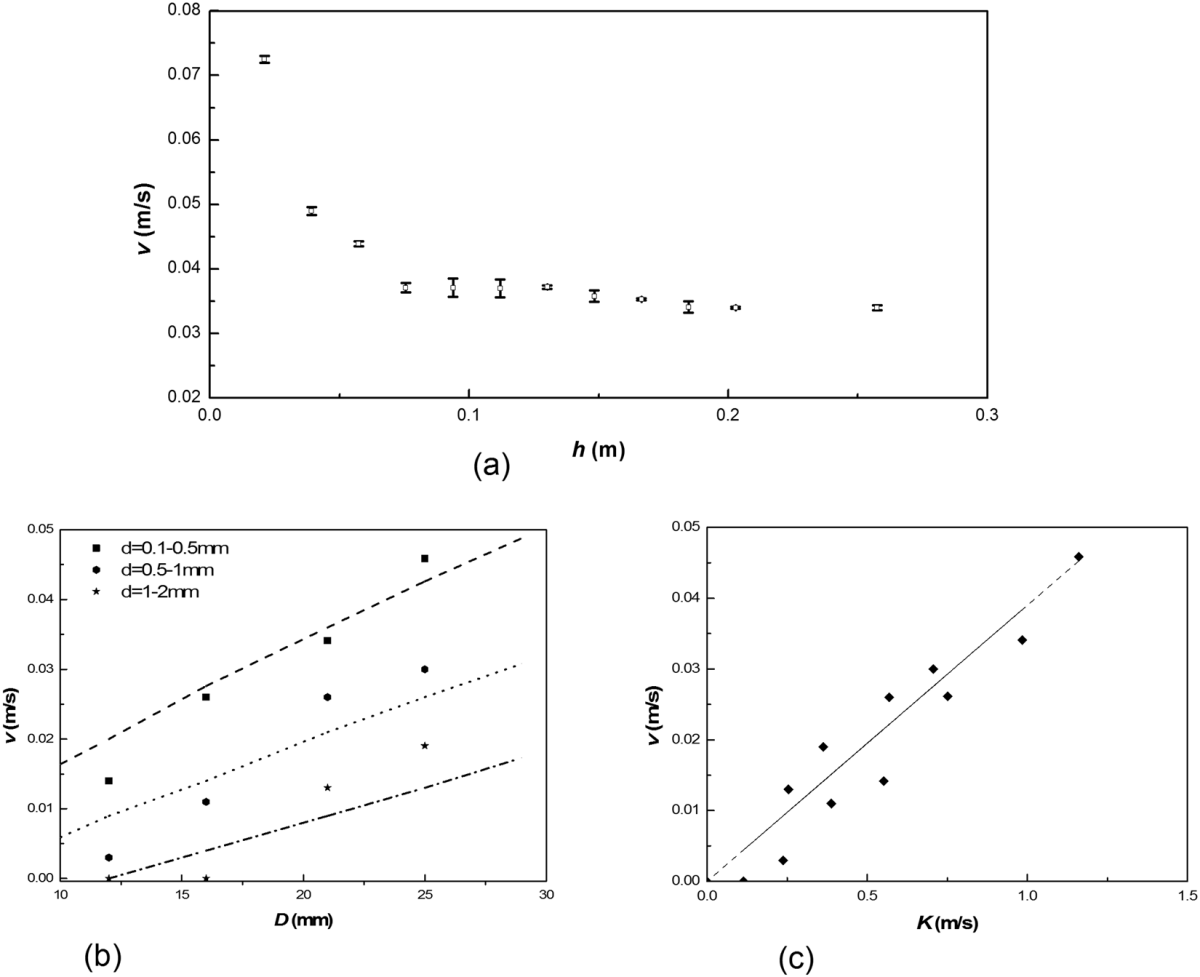
Relationship between *v* and *h*, *D* and *K*, where *K* is a function of the diameter of the beads (*D*) and sand (*d*).

Figure 3(a) shows the relationship between *v* and *h* for (*H*, *D*, *d*) = (800, 21, and 0.1 to 0.5) mm. It is noted that *v* decreases with *h* when the height of the porous bed is lower than a certain value, but approaches a constant value when the height of the porous bed is higher than this value. This implies that there is a critical height, beyond which the height of the porous bed has little influence on *v*. In this experiment, the value of *h* is about 0.1 m, and the ratio of *h* to *D* is about 5.

In the experiments, *H* and *h* are kept constant to investigate the relationship between *v* and *D* and *d*. Table 1 lists the results of 12 trials, which were all conducted with *H* = 0.8 m, *h* = 120 mm and a dry bulk density of sand of 1.57 g/cm^3^.

**Table 1:** Speed of sand flow *v* (mm/s) with different diameters of sand and beads.

*D* (mm)	25	21	16	12
*d* (mm)				
0.1–0.5	43.0	34.0	20.0	13.0
0.5–1.0	30.0	26.0	11.0	3.0
1.0–2.0	19.0	13.0	0.0	0.0

Eq. 1 is developed to fit *v* in this case,

1 $$v=a\sqrt{{\rm{g}}D}\,\mathrm{ln}(\frac{D}{kd})$$

where g is the acceleration of gravity; *D* is the diameter of the glass beads; *d* is the average grain size of the sand; *k* is the critical value related to the jamming phenomenon. When *D*/*d* is smaller than *k*, the sand is jammed by the porous bed. The coefficients *a* and *k* depend on the shape and roughness of the sand as well as the packed state of granular materials used in the experiment. For the sand and glass beads used in our research, *a* = 0.037 and *k* = 8, where the dry bulk density of the sand is 1.57 g/cm^3^, and the packed density of the glass beads is about 0.48.

Figure 3(b) shows the relationship between *v* and *D* for sands with different particle sizes, where the dotted curves are calculated from Eq. 1, while the points are experimental results listed in Table 1.

Figure 3(c) shows that *v* increases with an increase in *K*, where $K=\sqrt{gD}\,\mathrm{ln}(\frac{D}{8d}).$

### Simplified model of sand flow through porous bed

**Figure 4:**
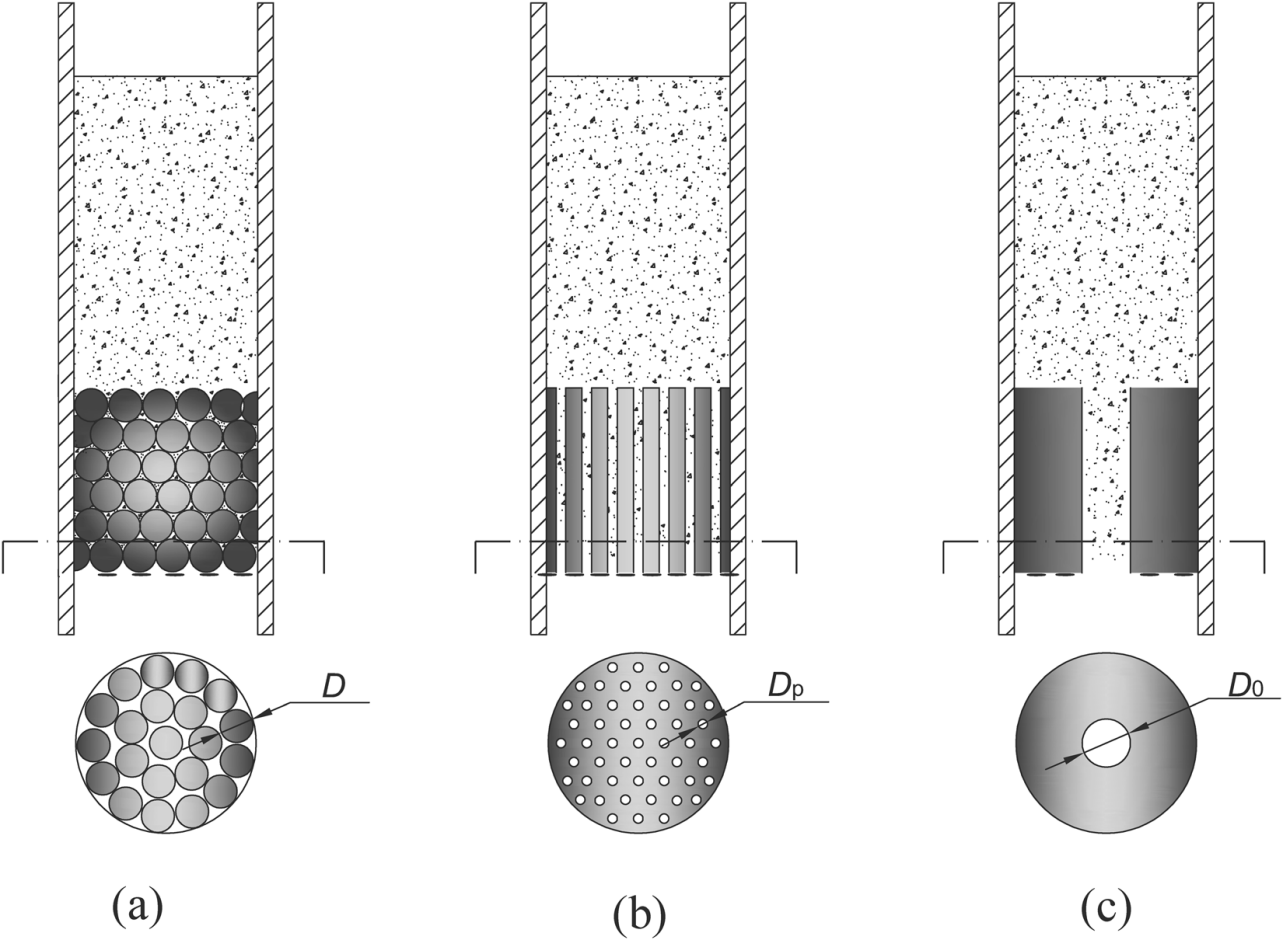
Schematic representation of sand flow through (**a**) porous bed; (**b**) multiple orifices; (**c**) one orifice.

In this study, we focused on the flow of fine sand through a porous bed (glass beads) with continuous voids. The flow rate of the sand mainly depends on the interstices of the pores. However, the interstices of the porous bed greatly vary both in shape and size^20^, depending on the different particle sizes of glass beads and arrangements. Since pore size is very challenging to describe, the calculation of the pore size is simplified and less complex. The porous medium shown in Fig. 4(a) can be simplified to parallel flow through a bundle of cylindrical pores or pipes with a fixed diameter (*D*_p_) as shown in Fig. 4(b). Furthermore, we can assume that the sand flow through a porous bed is like sand flow through an orifice with a diameter of *D*_0_, as shown in Fig. 4(c). Obviously, the values of *D*_p_ and *D*_0_ only depend on the interstices of the porous bed, but not on the grain size of sand.

### Parallel flow

The speed of sand flow through multiple pipes can also be expressed by using the drop distance of the sand per second, i.e. *v* = Δ*h*/*t*. If the flow rates of different sands through a porous bed are equal to those of sands through *N* multiple pipes which are uniformly distributed, then, these *N* multiple pipes with a diameter of *D*_p_ can be regarded as the equivalent multiple pipes of the porous bed. Therefore, it is expected that the speed is primarily related to the value of *D*_p_ and *N*.

**Figure 5:**
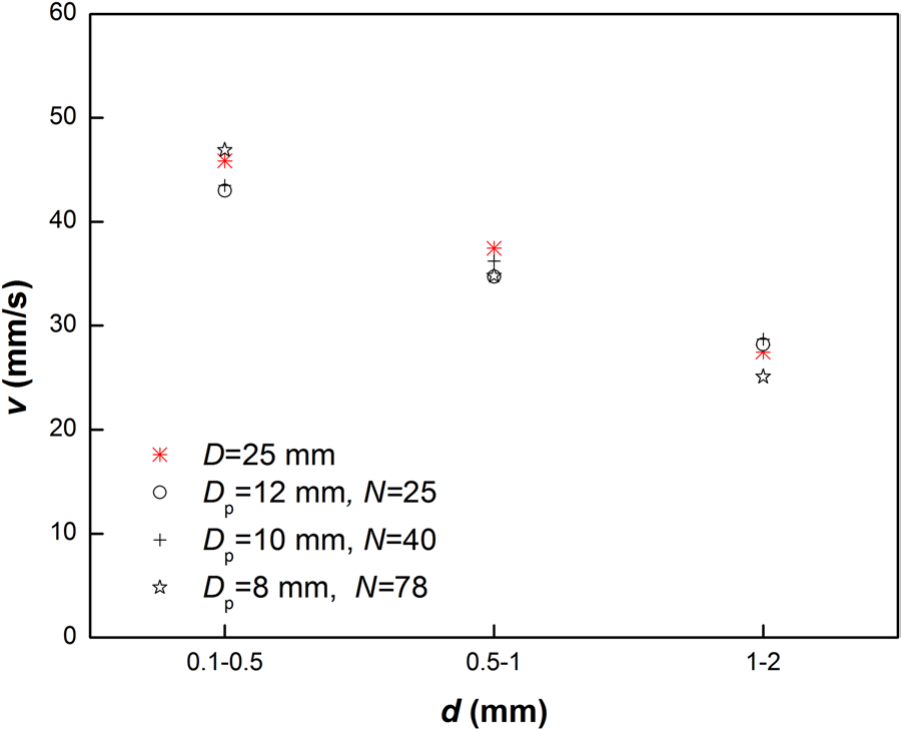
Equivalent pore diameter *D*_p_ and corresponding *N* for a porous bed with *D* = 25 mm.

We conducted several trials to identify the equivalent pore diameter of the porous medium by replacing circular plates with cylindrical pipes that have different diameters and adjusting the number of pipes (see Method section). Results show that there are a series of multiple pipes with *D*_p_ and *N*, through which the speed of the flow is equal to that of the sand flow through a porous bed. As an example, Fig. 5 shows the speed of flow for different grain sizes of sand through a porous bed with *D* = 25 mm. It is obvious that the speed of sand flow through these equivalent multiple pipes are approximately equal to that of sand flow through the porous bed. As expected, the measured mass flow rate for sand flow through these equivalent multiple pipes indicates a good agreement with that for sand flow through the porous bed.

Table 2 shows the series values of *D*_p_ and *N* for a porous bed with *D* = 25 mm. The relationship between *D* and *D*_p_ and *N* can be expressed as.

2 $${D}_{p}=C(1-e)D$$

3 $$e=\frac{{S}_{p}}{S-{S}_{p}}=\frac{N\pi {D}_{p}^{2}/4}{S-N\pi {D}_{p}^{2}/4}$$

where *e* is the void ratio by area, *S*_p_ is the total cross sectional area of the multiple pipes, and *S* is the area of the cylindrical tube bottom, and *C* is a coefficient which can be determined experimentally, *C* = 0.67 for *D* = 25 mm. *D*_p_ is less than *D*, since the porous bed cannot contain pores with a diameter greater than the diameter of the beads when they are packed. It is noted that the total cross sectional area with a larger *D*_p_ and less *N* are greater than that with smaller *D*_p_ and more *N*.

**Table 2:** Equivalent diameter *D*_p_ and number *N* of parallel pipes for a porous bed with *D* = 25 mm.

*D*_p_ (mm)	12	10	8
*N*	25	40	78
Total cross sectional area of pipes (mm^2^)	2826.0	3140.0	3918.7

### Beverloo’s law

According to Beverloo’s law, the mass flow rate through an orifice is determined with:

4 $$W={C}_{0}{\rho }_{{\rm{b}}}\sqrt{{\rm{g}}}{({D}_{0}-{k}_{0}d)}^{5/2}$$

where *W* is the average mass discharge rate through an orifice; *C*_0_ and *k*_0_ are the empirical discharge and shape coefficients, respectively; *ρ*_b_ is the apparent density; g is the acceleration of gravity; *D*_0_ is the diameter of the outlet of an orifice; and *d* is the diameter of the particles. We conducted an experiment with sand flowing through an orifice and determined the value of *C*_0_ and *k*_0_. In this study, *C*_0_ = 0.5 and *k*_0_ = 1.26 for sand with a grain size that ranges from 0.1 to 0.5 mm; *C*_0_ = 0.5 and *k*_0_ = 1.3 for 0.5 to 1 mm; and *C*_0_ = 0.5 and *k*_0_ = 1.4 for 1 to 2 mm.

We assumed that the sand flow through a porous bed (Fig. 4(a)) is like sand flow through an orifice with a diameter of *D*_0_ (Fig. 4(c)). Table 3 lists *D*_0_ for different porous beds and sands calculated according to Beverloo’s law and the measured mass flow rate (raw data provided in Supplementary Information). Since *D*_0_ does not depend on the grain size of sand, there should be only one *D*_0_ for one porous bed in case that there is the same mass flow rate while sand flow through the porous bed and the orifice, as mentioned above. However, the value of *D*_0_ is not a constant value, but varies with the grain size of sand for the same porous bed (Table 3). For example, *D*_0_ ranges from 30 to 40 mm, 25 to 36 mm, 24 to 30 mm, and 15 to 25 mm for a porous bed with *D* = 25, 21, 16, and 12 mm, respectively. This indicates that the sand flow through a porous bed cannot be simplified to be the same as sand flow through an orifice with a certain diameter. In addition, with respect to the flow pattern and boundary conditions, there is a significant difference between the sand flow through an orifice and that through a porous bed. The dropping top surface of the sand flow through multiple parallel orifices and a porous bed may be flat, while it changes to a hopper shape when sand flow through one orifice.

**Table 3:** Calculated value of *D*_0_ (mm) with different diameters of sand and beads.

*D* (mm)	25	21	16	12
*d* (mm)				
0.1–0.5	39.3	36.3	29.5	24.8
0.5–1.0	35.2	33.3	24.0	14.6
1.0–2.0	30.6	26.6		

If the sand flow through multiple pipes (Fig. 4(b)) can also be regarded as the sand flow through an orifice (Fig. 4(c)), then the total mass flow rate (*W*_p_) could be obtained by Eq. (5).

5 $${W}_{p}=N\,{C}_{0}{\rho }_{{\rm{b}}}\sqrt{{\rm{g}}}{({D}_{p}-{k}_{0}d)}^{5/2}$$

**Figure 6:**
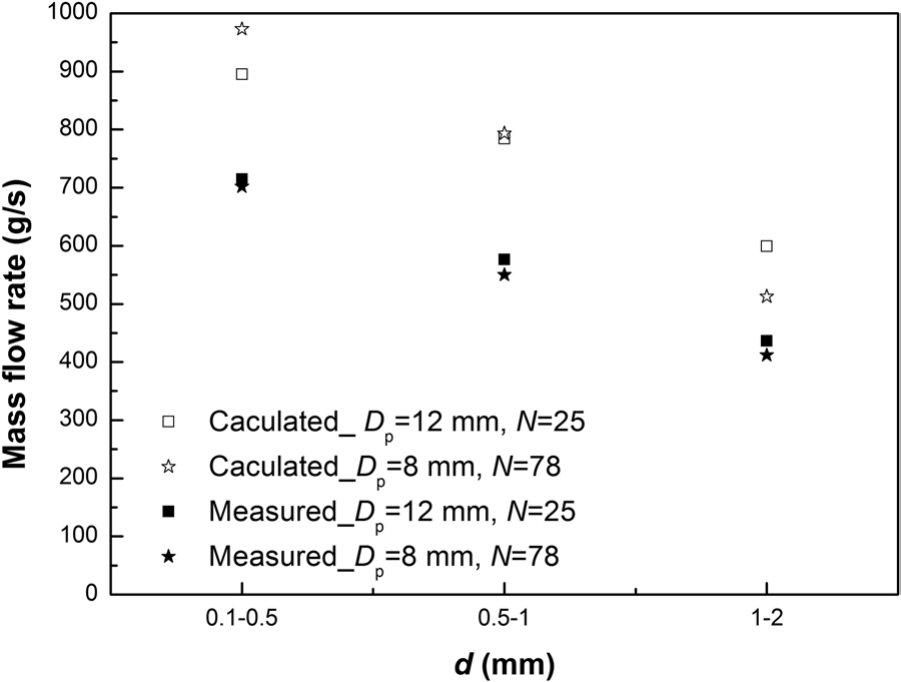
Comparison of measured mass flow rate through equivalent multiple pipes and the ones calculated from Beverloo’s law.

However, the measured mass flow rates through these equivalent multiple pipes do not match the calculated ones by using Beverloo’s law. Figure 6 shows a comparison between the measured ones and the calculated ones. This also implies that the sand flow through multiple pipes cannot be simplified to be the same as sand flow through an orifice with a certain diameter.

## Discussion

We examined the speed of sand flow through a porous bed under various conditions, including the height of the porous bed (*h*) and sand column (*H*), and the diameter of the glass beads (*D*) and sand (*d*). The relationship between the speed of the sand flow (*v*) and these different conditions was investigated with glass beads packed at a constant density of 0.48 and sand with a dry bulk density of 1.57 g/cm^3^. We found that the speed of the sand flow (*v*) depends on all of these factors except for *H*. The speed of the sand flow (*v*) decreases with height of the porous bed (*h*) when the latter is lower than a certain value. However, if *h* is higher than this value, *v* is approximately constant, and the relationship between *v* and *D*, *d* can be expressed in terms of $v=a\sqrt{{\rm{g}}D}\,\mathrm{ln}(\frac{D}{kd})$, where g is the acceleration of gravity and *a* and *k* are coefficients, which should be determined through the use of experiments.

A porous bed can be modeled as a cylindrical pore model that consists of a number of cylindrical pipes; therefore, the sand flow through a porous bed could be assumed as parallel flow. Here, we discuss the relationship between the parameters of the cylindrical pore model (*D*_p_, *N*) and the diameter of glass beads (*D*) in a porous bed. The experimental results showed that there can be different equivalent pore diameters with different corresponding number of pores or pipes.

Additionally, we determined the coefficients of *C*_0_ and *k*_0_ for sand flow through an orifice by using Beverloo’s law through experiments. We attempted to find the *D*_0_ by using Beverloo’s law to match the measured mass flow rate (*W*) of sand flow through a porous bed. However, we found that the value of *D*_0_ is not absolute, which means that the calculated *D*_0_ is different for sand with different particle sizes that flow through the same porous bed. This implies that a porous bed cannot be simply assumed to be the same as an orifice with a certain diameter. The reason for this needs further study. Maybe because there exist obstructions among the sand particles and glass beads when sand flows through the porous bed while Beverloo’s law was drawn from experiments of granular material flow through an orifice without other obstructions.

Many geological processes, such as landslides, erosion, and debris flow, involve granular flow. Our findings add to the existing formulas on granular flow with an expression of sand that flows through a fixed granular bed by using an orifice proposed in Beverloo et al. and Hersam^8, 10^. Moreover, the result also provides a better understanding on the mechanisms of sand flow through a porous bed, which is helpful for preventing flow hazards in the industry or geological hazards in geo-engineering. For example, the design of a grill placed in front of a dam requires knowledge of the grain size distribution of the solids in the debris flow. Moreover, the results have significance in preventing natural geological hazards in mining since jamming could take place when the ratio of the rock size in the caving zone to the overburden sand is small enough (see Supplementary Information).

However, there are some limitations and several issues that need to be addressed in further studies. For example, there is the need to theoretically analyze why the height of the sand has little effect on the speed of the sand flow through granular material. Moreover, this study has only investigated the sand flow through a packed porous bed (by using glass beads) in various conditions, and the coefficients *a* and *k* were determined by using only four factors (*D*, *d*, *h* and *H*). There is no doubt that different dry bulk densities of sand and different particle arrangements in the porous bed influence the speed of the sand flow. Therefore, further experiments need to be carried out that investigate the coefficients *a* and *k* with different grain size distributions, dry bulk densities of sand, porous beds with different particle arrangements, and roughness and shape of granular matters. Especially, further study is needed for investigating the result that the flow of sand through a porous bed or multiple parallel pipes cannot be simplified to flow through one orifice with a certain diameter.

## Methods

### Experiment: sand flow through porous bed

The experiments were performed in a cylindrical tube with a height of 2000 mm and diameter of 120 mm. The glass beads have four different diameters of 25 mm, 21 mm, 16 mm, and 12 mm, and fixed in place with chicken wire. The glass beads were placed into the cylindrical tube in layers, with each layer closely packed a density of approximately 0.48. A bulkhead was placed above the top of the glass beads, so that the sand would instantaneously flow into the porous bed of glass beads when the plate was removed after the start of the experiment. The sand used in these experiments is natural river sand sampled from Xuzhou in Jiangsu Province, China. The majority of the grains of sand are sub-angular in shape. The sand was washed and then sieved. Sand with three different particle sizes was used: 0.1–0.5 mm, 0.5–1.0 mm, and 1.0–2.0 mm, respectively. The direct shear tests showed that their corresponding internal angle of friction is 40°, 41.1° and 43.3°, respectively. In the preparation of the sand samples, the dry bulk density was maintained as 1.57 g/cm^3^. The amount of sand required was determined so as to correspond to this dry bulk density.

In the first experiment, we focused on investigating the effect of the height of the glass beads on the flow rate, with all other variables held constant. The diameter of the glass beads is 21 mm, and the particle size of the sand ranges from 0.1 to 0.5 mm. The results showed that changes in the speed of the sand flow is minimal if the height of the glass beads is over 105 mm. This phenomenon was taken into consideration so that in the second experiment, the height of the glass beads was set to be more than 120 mm, and glass beads and sand that have different diameters were used. Consequently, the influence of the grain size on the speed of the flow could be investigated.

### Experiment: sand flow through parallel pipes

The experiment was performed in a cylindrical tube with a height of 2000 mm and diameter of 120 mm. A flat circular plate and parallel cylindrical pipes were placed on the base of the cylindrical tube. The length of the pipes was set to be 120 mm, which is the same as the height of the porous bed. The diameter of the pipes ranged from 5 to 12 mm. A box was used to collect sand from the outlet of the pipe, and placed onto an electronic scale, from which the weight of the sand flow was obtained. Since the mass flow rate does not depend on the height of the sand column, the latter was set to 0.5 m. Sand with a grain size that ranged from 0.5 to 1 mm and 1 to 2 mm was used. The dry bulk density of the sand was 1.57 g/cm^3^.

In the experiments, we used circular plates with different values of *D*_p_ and *N* to fit the mass flow rate through the porous bed. When the speed of the sand flow through *N* multiple pipes is equal to that through the porous bed, the diameter of a pipe (*D*_p_) can be regarded as having the equivalent pore diameter of the porous bed, and the number of pipes were denoted as *N*.

### Experiment: sand flow through orifice

This experiment was carried out in a cylindrical tube with a height of 2000 mm and diameter of 120 mm, and a flat circular plate with circular orifices *D*_0_ was placed on the base of the cylindrical tube. This plate with orifices that have different outlets could be removed and replaced with another, and therefore allowed us to modify the diameter of *D*_0_, which ranged from 20 to 50 mm*.* At the bottom of the cylindrical tube, a box collected the sand from the outlet of the pipe. This box was then placed on an electronic scale, from which the weight of the sand flow was obtained. Previous experimental data were used, and in order to do so, sand with grain size that ranged from 0.5 to 1 mm and 1 to 2 mm was used, and the dry bulk density of sand was maintained at 1.57 g/cm^3^. Since the mass flow rate does not depend on the height of the sand column, the latter is set to 0.5 m.

Circular orifices *D*_0_ with a diameter of 50, 40, 30 and 20 mm were used. The aim was to determine the coefficients of Beverloo’s equation for the sand used in the study under the same condition.

## Electronic supplementary material

Supplementary Video S1

Supplementary Video S2

Supplementary Video S3

Supplementary information
