# Supplementary material for: An experimental investigation on the speed of sand flow through a fixed porous bed
Source: Sci Rep. 2017 Mar 3;7:54. doi: 10.1038/s41598-017-00082-2 (PMC5428362; doi:10.1038/s41598-017-00082-2)
Supplement: Supplementary file 4 — Supplementary information [file 41598_2017_82_MOESM4_ESM.pdf]

An experimental investigation on the speed of sand flow through a fixed porous bed

Wanghua Sui[1*], Yankun Liang[1*], Xinjia Zhang[1], Ravi Jain[2], Tao Zhu[3]

[1] *School of Resources and Geosciences, China University of Mining and Technology, Xuzhou, Jiangsu, China.*

[2] *School of Engineering and Computer Science, University of the Pacific, California, USA.*

[3]*WanliCoalmine No.1, Shenhua Shendong Group Co. Ltd., Ordos, Inner Mongolia, China*

E-mail: suiwanghua@cumt.edu.cn

*These authors contributed equally to this work.

Supplementary information

Videos S1 to S3 show the process of sand flow passing through the granular bed in the experiments under different initial conditions.

Supplementary Video S1 In this video, the height of fixed granular bed *h* is set to be 120 mm, the diameter of the glass beads *D* is 12 mm, and the particle size of sand is 0.5-1.0 mm. The speed of the sand flow is about 30 mm/s.

Supplementary Video S2 In this video, sands with a particle size of 0.1-0.5 mm flow through a granular bed with a diameter *D* of 21 mm; the height of fixed granular bed *h* is set to be 126 mm. The speed of the sand flow is about 34 mm/s.

Supplementary Video S3 The height of fixed granular bed *h* is 126 mm, the diameter of the glass beads *D* is 12 mm, and the diameter of sand is 0.5-1.0 mm. The speed of the sand flow is about 26 mm/s.

Supplementary information_1: an example of sand flow hazard through caving zone.

Supplementary information_2: experimental results of sand flow through porous bed, parallel pipes and orifices.

Supplementary information -1

Supplementary: an example of sand flow hazard through caving zone

The mining of shallow seams is very common in western China, in which the depth of the coal seam is usually 200m. Alluvium aquifers are also directly found above the shallow coal seams. During mining, mining-induced overburden failure develops on the ground surface, which could result in serious geological hazards because aeolian sand flows through the broken rock, caving zone or the rock mass fractures during coal mining (**Figure S1-1**). In 2010, aeolian sand flowed into the caving zone in Panel 22402 of the Halagou Coalmine, and caused surface subsidence[1].Tocontrol the aeolian sand flow, the mining staff threw many sandbags and geo-textiles into the subsided basin. In the end, the sand flow was jammed, and a funnel-shaped collapse with a diameter of 47m and depth of 12m formed on the ground surface (**Figure S1-2**).

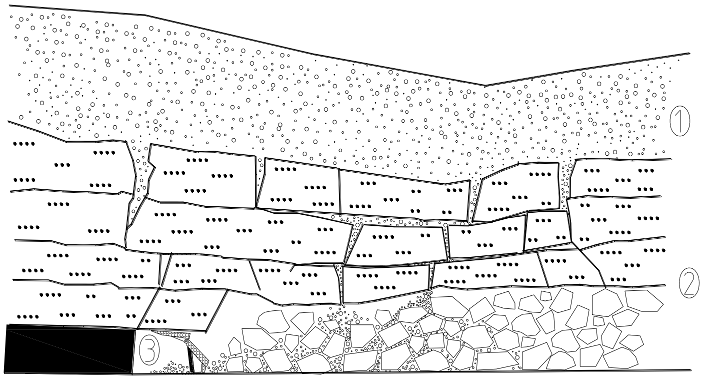

1 - Aeolian sand; 2 - Fractured overburden3 - Working face in panel

Figure S1-1. Engineering geological model of mining under sand

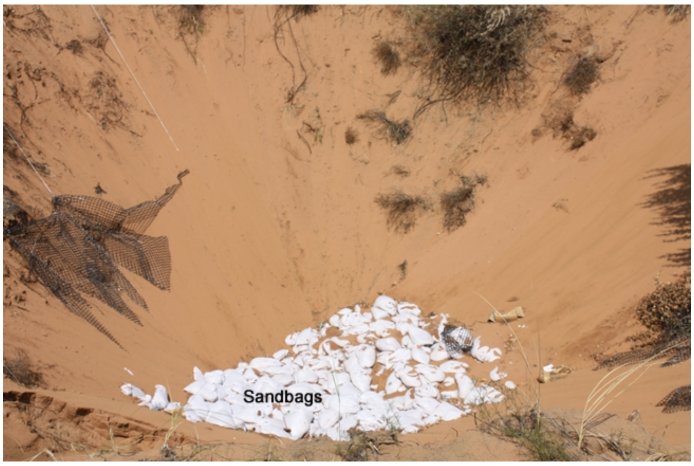

Figure S1-2. Subsided sinkhole caused by sand flow into underground panel

Reference

1. Song Y. Water inrush and sand inrush mechanism and prevention technology during the initialmining period in 22402 working face of Halagou coal mine. Coalmine Safety. 43(12): 91-93 (2012)

Supplementary information -2

Supplementary: primary data of experiments

Table 1 and **2** are the primary data of 12 experiments of sand flow through porous bed. *H*, *h*, *D*, *d* denote the height of sand column, the height of porous, the diameter of glass bed and the grain size of sand.

Table 3 and **4** are primary data of sand flow through multiple pipes with different number and diameters (N, D_p).

Table 5 is the measured mass flow rate (g/s) with different diameters of sand and beads

Table 1 The position of free surface vs time for *D*=25 and 21 mm (*H*=500 mm， *h*=120 mm)

t(s)	The drop distance of the sand surface (mm)					
	D=25 mm			*D*=21 mm		
	d=0.1-0.5	*d*=0.5-1.0	*d*=1.0-2.0	*d*=0.1-0.5	*d*=0.5-1.0	*d*=1.0-2.0
0	0	0	0	0	0	0
2	110	100	60	85	75	60
3	155	140	80	120	100	80
4	200	170	100	155	130	95
5	245	200	120	190	160	110
6	290	230	140	225	190	125
7	340	260	160	260	210	140
8	380	290	180	290	240	155
9	430	320	200	330	270	170
10	465	350	220	360	300	185
11	500	380	240	395	315	200
12		410	260	430	340	215
13		440	280	465	370	230
14		470	290	500	395	245
15		500	310		402	260
16			330		440	275
17			350		470	290
18			370		500	300
19			380			310
20			400			320
21			420			330
22			440			345
23			460			360
24			480			370
25			500			385
27						410
31						435
33						460
34						500

Table 2 The position of free surface vs time for *D*=16 and 12 mm. (*H*=500 mm, *h*=120 mm)

t(s)	The drop distance of the sand surface (mm)					
	D=16 mm			*D*=12 mm		
	d=0.1-0.5	*d*=0.5-1.0	*d*=1.0-2.0	*d*=0.1-0.5	*d*=0.5-1.0	*d*=1.0-2.0
0	0	0	0	0	0	0
2	750	70	0	25	20	0
3	100	80	0	45	30	0
4	120	100	0	6	35	0
5	140	120	0	75	40	0
6	160	130	0	90	45	0
7	180	140	0	105	50	0
8	200	150	0	120	50	0
9	220	170	0	130	55	0
10	240	180	0	145	60	0
11	260	190	0	160	65	0
12	280	210	0	170	70	0
14	320	240	0	200	75	0
15	340	250	0	210	80	0
17	380	270	0	240	85	0
18	400	280	0	255	90	0
20	440	310	0	280	95	0
22	480	340	0	300	100	0
23	500	350	0	310	100	0
24		360	0	320	105	0
26		380	0	350	110	0
28		410	0	375	115	0
29		420	0	390	120	0
31		440	0	420	125	0
32		450	0	430	125	0
33		460	0	440	130	0
36		490	0	480	135	0
37		500	0	495	135	0
42			0		145	0
50			0		165	0
55			0		180	0

Table 3 The position of free surface vs time for different N and D_p (H=500 mm，h=120 mm)

t(s)	The drop distance of the sand surface (mm)					
	N=35, D_p=12 mm			N=40, D_p=10 mm		
	d=0.1-0.5	d=0.5-1.0	d=1.0-2.0	d=0.1-0.5	d=0.5-1.0	d=1.0-2.0
0	0	0	0	0	0	0
1	45	35	27	47	36	30
2	87	70	55	92	71	55
3	132	104	85	137	101	85
4	177	137	115	179	141	110
5	218	170	144	225	176	140
6	260	20.4	170	227	216	166
7	302	23.9	200	272	251	195
8	341	277	227	317	326	220
9	380	313	252	367	361	250
10			280			278

Table 4 The position of free surface vs time for different N and D_p (H=500 mm，h=120 mm)

t(s)	The drop distance of the sand surface (mm)		
	N=78, D_p=8 mm		
	d=0.1-0.5	d=0.5-1.0	d=1.0-2.0
0	0	0	0
1	50	35	25
2	97	66	50
3	145	105	75
4	190	140	100
5	235	175	123
6	280	207	148
7	325	245	175
8			199
9			225
10			250

Table 5 Average mass flow rate (g/s) with different diameters of sand and beads

d (mm) \ *D* (mm)	25	21	16	12
0.1 - 0.5	736.0	566.6	434.5	236.0
0.5 - 1.0	506.0	425.4	182.8	49.0
1.0 - 2.0	339.8	227.7	0.0	0.0
